# Downregulation of miR-1-3p expression inhibits the hypertrophy and mineralization of chondrocytes in DDH

**DOI:** 10.1186/s13018-021-02666-1

**Published:** 2021-08-18

**Authors:** Rui Ding, Xijuan Liu, Jian Zhang, Jinghong Yuan, Sikuan Zheng, Xigao Cheng, Jingyu Jia

**Affiliations:** 1 Department of Orthopedics, The Second Affiliated Hospital of Nanchang University, No. 1 Minde Road, Donghu District, Nanchang, Jiangxi China; 2 Department of Pediatrics, The Second Affiliated Hospital of Nanchang University, No. 1 Minde Road, Donghu District, Nanchang, Jiangxi China; 3 Institute of Orthopedics of Jiangxi Province, Nanchang, Jiangxi China; 4 Institute of Minimally Invasive Orthopedics of Nanchang University, Nanchang, Jiangxi China

**Keywords:** Developmental dysplasia of the hip, MicroRNA, miR-1-3p, SOX9, Endochondral ossification

## Abstract

**Background:**

Developmental dysplasia of the hip (DDH) is a highly prevalent hip disease among children. However, its pathogenesis remains unclear. MicroRNAs (miRNA) are important regulators of cartilage development. In a previous study, high-throughput miRNA sequencing of tissue samples from an animal model of DDH showed a low level of miR-1-3p in the cartilage of the acetabular roof (ARC), but its role in DDH pathogenesis was not addressed. Therefore, our aim here was to investigate the effects of miR-1-3p in the ARC.

**Methods:**

The diagnosis of acetabular dysplasia was confirmed with X-ray examination, while imaging and HE staining were conducted to further evaluate the ARC thickness in each animal model. FISH was employed to verify miR-1-3p expression in the ARC and chondrocytes. The miR-1-3p target genes were predicted by a bioinformatics database. A dual-luciferase reporter assay was used to confirm the targeting relationship between miR-1-3p and SOX9. The gene expression of miR-1-3p, SOX9, RUNX2 and collagen type X was evaluated by qPCR analysis. The protein expression of SOX9, RUNX2 and collagen type X was detected by western blot analysis. The levels of SOX9, RUNX2, and collagen type X in the ARC were further assessed via immunohistochemistry analysis. Finally, Alizarin Red S staining was used to observe the mineralized nodules produced by the chondrocytes.

**Results:**

We observed low expression of miR-1-3p in the ARC of animals with DDH. SOX9 is a miR-1-3p target gene. Using miR-1-3p silencing technology in vitro, we demonstrated significantly reduced chondrocyte-generated mineralized nodules compared to those of the control. We also confirmed that with miR-1-3p silencing, SOX9 expression was upregulated, whereas the expression of genes associated with endochondral osteogenesis such as RUNX2 and collagen type X was downregulated. To confirm the involvement of miR-1-3p silencing in abnormal ossification through SOX9, we also performed a rescue experiment in which SOX9 silencing restored the low expression of RUNX2 and collagen type X produced by downregulated miR-1-3p expression. Finally, the elevated SOX9 levels and reduced RUNX2 and collagen type X levels in the ARC of rabbits with DDH were also verified using immunohistochemistry, RT-PCR, and western blots.

**Conclusion:**

The relatively low expression of miR-1-3p in the ARC may be the cause of abnormal endochondral ossification in the acetabular roof of animals with DDH.

## Introduction

Developmental dysplasia of the hip (DDH) is a highly prevalent three-dimensional deformity among children [1]. Despite prompt treatment, either conservatively or surgically, some infants and children can still develop residual acetabular dysplasia, which can progress to severe osteoarthritis in adulthood, resulting in the need for total hip replacement [2, 3]. In children, the acetabulum is mainly composed of the ilium, ischium, pubis, and Y-shaped epiphyseal plate (triradiate cartilage), wherein the ilium forms the acetabular roof and the pubis and ischium constitute the anterior and posterior walls of the acetabulum, respectively [4–6]. Using the 3DCT split technique, we found developmental defects on the lateral side of the acetabular roof and the acetabular index increased in children with untreated DDH. In addition, the ischium was rotated, promoting increased forward inclination of the acetabulum [4, 5]. In treated children with DDH, however, in a period of 10–59 months, we found that all structural defects, namely, developmental defects on the anterior wall of the acetabulum, thickening of the posterior and medial walls, rotation of the ischium, and forward inclination of the acetabulum, returned to normal. However, some children still exhibited developmental defects in the acetabular roof, and the tilt angle remained high [7]. In work done by Mootha et al. [8] involving 45 children with DDH aged 12–48 months, it was also shown that acetabular anteversion was increased in the children with DDH relative to healthy aged-matched children. This finding was consistent with other studies that also showed a marked association between developmental defects of the acetabular roof and DDH [9, 10].

To reveal the pathogenesis behind acetabular roof developmental defects in children with DDH, we established a DDH animal model by reported methods, e.g., straightening and swaddling the legs of New Zealand white rabbits [11, 12]. We observed that the acetabular roof cartilage (ARC) was thicker on the side where the limb was straightened, e.g., the DDH side compared to the untreated control side. This phenomenon was also reported by Li et al. [12]. Our MRI results were also similar to MRI observations of children with DDH [13]. Moreover, in our previous study, we confirmed that abnormal endochondral ossification is the cause of the thickness of acetabular roof cartilage in DDH models [13].

MiRNAs are 21–24 nucleotide long, highly conserved, noncoding (nc) RNA molecules. These molecules function by base pairing with the 3′-noncoding region of the target mRNA molecules to suppress translation and drive degradation of the mRNAs [14]. According to bioinformatics studies, approximately one-third of all human mRNAs are subject to regulation by miRNAs [15]. Recent evidence suggests that the abnormal expression of miRNAs is strongly associated with the proliferation, differentiation, apoptosis, and development of chondrocytes [16–20]. To examine the role of miRNA in the thickening of the acetabular roof cartilage in DDH, researchers examined differential miRNA expression in the acetabular roof cartilage of DDH models versus untreated controls. Based on the results from the miRNA microarray, 18 miRNAs were differentially expressed: 3 highly expressed miRNAs and 15 poorly expressed miRNAs [13]. In subsequent experiments, it was confirmed that the miRNA miR-129-5p regulated GDF11 expression to modulate Smad3/IHH-induced endochondral ossification in the acetabular cartilage of a DDH model [13].

To further elucidate the role of other miRNAs in DDH pathogenesis, we performed qPCR verification and discovered very low expression of miR-1-3p, which was in accordance with our previous miRNA chip data [13]. It has been reported that miR-1-3p expression is downregulated in osteoarthritic cartilage and confirmed that miR-1-3p is closely related to osteoarthritis [21]. However, the role of miRNA in DDH is unknown. This study aimed to explore the miR-1-3p levels in acetabular roof cartilage and examine its underlying mechanism of action in the pathogenesis of DDH.

## Materials and methods

### Establishment of the experimental animal model of DDH

**Fig. 1:**
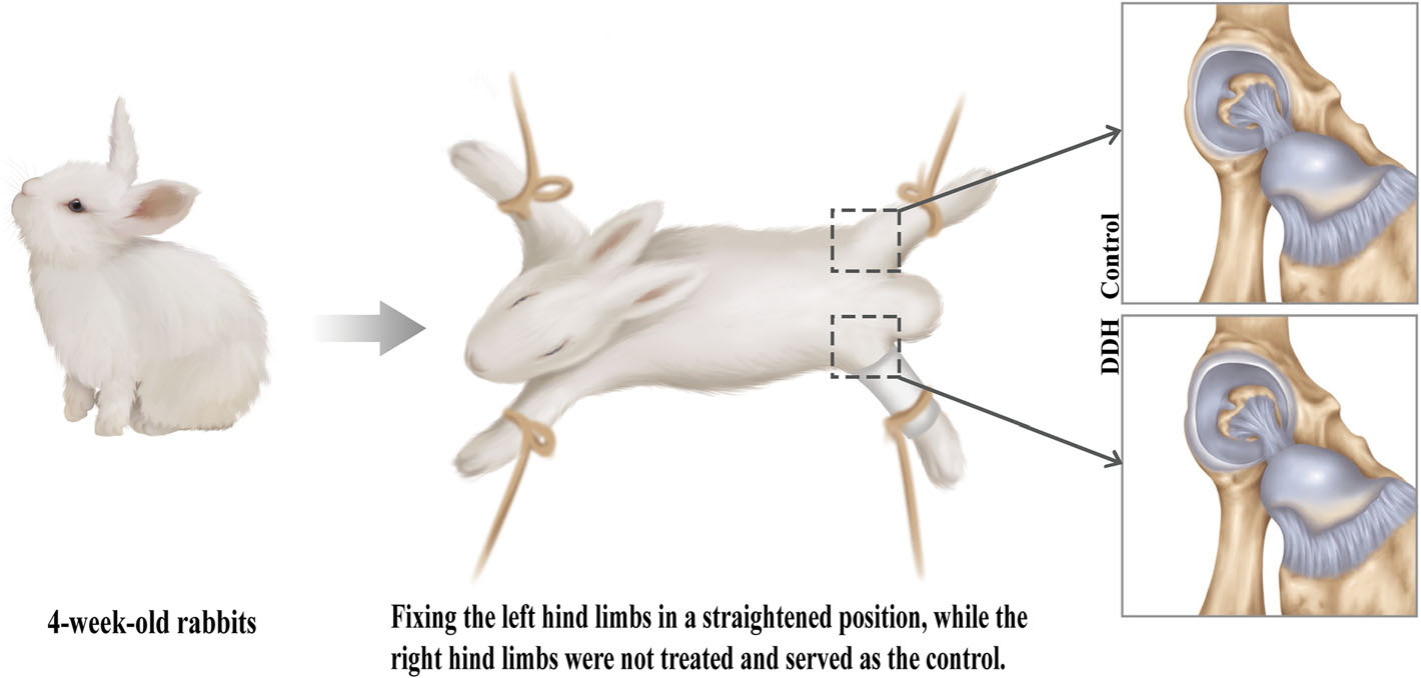
Schematic of the established DDH rabbit model. Thirty 4-week-old rabbits were used to induce DDH by fixing the left hind limbs in a straight position, while the right hind limbs were left untreated and served as controls. The rabbits were given free access to food and water until 8 weeks of age, at which time the rabbit was euthanized for examination of the ARC. The ARC was found to be significantly thicker in the rabbits with DDH, as compared to the controls

Thirty 4-week-old, healthy, New Zealand white rabbits (Nanchang Longping Domestic Rabbit Industry, Jiangxi Province, China), weighing 430–640 g, were used to induce DDH by fixing the left hind limbs in a straight position, while the right hind limbs were left untreated to serve as controls (Fig. 1). The animals were fed and maintained in a room with a temperature of 23 °C and 50% humidity. The room was cleaned every other day. New feed (purchased from Lanling Hekangyuan Feed, China) and purified water were added to cages every 2 days to feed the rabbits until 8 weeks of age. At 8 weeks of age, X-ray examination was performed to confirm acetabular dysplasia using the previous X-ray indicators [22], and MRI imaging was conducted to further evaluate the ARC thickness in each animal model. The evaluations of acetabular dysplasia and ARC thickness were carried out by two individual orthopedic physicians. Statistical analysis was performed by other researchers. These evaluations adopted a single-blinded design; i.e., those evaluating the data were not informed which data came from which experimental group. This study was approved by the Ethics Committee of the Second Affiliated Hospital of Nanchang University in Jiangxi Province, China ([2017] No. (091)).

### Hematoxylin and eosin staining

The hip joint specimens were placed in 10% EDTA for decalcification for 4 weeks. Next, the specimens were cut to expose the coronal surface of the hip joint, as depicted in Fig. 1B (red line), and embedded in paraffin. The paraffin-embedded specimens were then cut into 4-μm-thick sections, placed on glass slides, dewaxed in xylene solution, hydrated, and stained with hematoxylin dye for 1 min. Next, the sections were washed and placed in hydrochloric acid alcohol solution for differentiation, followed by bluing in Scott’s solution for 3 min, washing, and staining with eosin dye for 10 min. The sections were then dehydrated in graded ethanol solutions and mounted with neutral resin, followed by overnight (O/N) drying at 65°. Finally, the stained sections were photographed under a light microscope at × 100 magnification, and the cartilage thickness was measured using Image-Pro Plus 6.0 software (Media Cybernetics, Inc., Rockville, MD, USA).

### Bioinformatics prediction

TargetScan (http://www.targetscan.org/vert_71/) was used to identify downstream target genes of miR-1-3p.

### Dual-luciferase reporter gene assay

MiR-1-3p mimic (WT), miR-1-3p mutant (MUT), and negative control (NC) were cotransfected into human primary chondrocytes together with the Sox9 3′UTR luciferase reporter plasmid (GenScript) and Renilla reporter control. After 48 h, the cells were harvested using a Dual-Lumi™ II Luciferase Reporter Assay kit (Beyotime Institute of Biotechnology, Shanghai, China), in accordance with the manufacturer’s instructions, and the luciferase activity was detected using a multimode reader (BioTek Instruments, Inc.). The firefly luciferase activity was normalized using Renilla luciferase activity.

### Chondrocyte culture

The ARC was chopped and digested with 0.25% trypsin at 37 °C for 30 min, followed by culturing in serum-free DMEM with 0.2% type II collagenase for 4 h. The isolated rabbit chondrocytes were subsequently subcultured in complete DMEM (Gibco, Waltham, MA, USA) with 10% fetal bovine serum (FBS) and 1% penicillin/streptomycin (PS) in a humid environment with 5% CO2 at 37 °C for future experiments. Human primary chondrocytes were purchased from Procell Life Science & Technology Co., Ltd. (Wuhan, Hubei Province, China) were cultured in complete DMEM with 10% FBS and 1% PS in an incubator with 5% CO2 at 37 °C.

### Transfection

At 80–90% confluency, miR-1-3p inhibitor, NC-miRNA inhibitor, SOX9 siRNA, or siNC messenger RNA (mRNA) (RiboBio, Guangzhou, Guangdong Province, China) was transfected into human primary chondrocytes using Lipofectamine 3000 reagent following the manufacturer’s guidelines (Invitrogen, Carlsbad, CA, USA) and cultured in serum-free DMEM for 6 h, followed by culturing in DMEM with serum for 72 h. These cells were next used for further experiments.

### RNA extraction and qPCR

Total RNA was isolated from either the ARCs of 7 rabbits or cultured chondrocytes using TRIzol reagent (Invitrogen, Waltham, MA, USA). Next, the RNA samples were reverse transcribed using the PrimeScript™ RT reagent Kit with gDNA Eraser kit (TaKaRa Bio, Kusatsu, Japan). The extracted total RNA was then quantified using the ABIQ6PCR system. The miRNA primers were designed by RiboBio (Guangzhou, Guangdong Province, China) and are summarized in Table 1. All experiments were performed 3×, and the mean value of the results is presented in this paper.

**Table 1:** Primer sequence for real-time quantitative PCR

Gene	Forward	Reverse
miR-1-3p	ACACTCCAGGTGGGTGGAATGT	CTCAACTGGTGTCGTGGAG
U6	CTCGCTTCGGCAGCACA	AACGCTTCACGAATTTGCGT
SOX9	GGCAAGCTCTGGAGACTTCTG	CTGCCCATTCTTCACCGACTT
RUNX2	GAATGCTTCATTCGCCTCACA	TGGCTGGATAGTGCATTCGT
COL10	CTCGTGGAAATGATGGTGCT	ACCAGGTTCACCGCTGTTAC
GAPDH	GGGCAGAGGAAGCTTCAGAAA	TCTCAGATGGATTCTGCGTGC

### Western blotting

Total protein was isolated from either the ARC of 7 rabbits or cultured chondrocytes using RIPA lysis buffer (Applygen Technologies, Inc., Beijing, China) and quantified using a bicinchoninic acid (BCA) protein assay kit (Pierce Biotechnology, Rockford, IL, USA), followed by separation using sodium dodecyl sulphate polyacrylamide gel electrophoresis (SDS-PAGE) and subsequent transfer of proteins to polyvinylidene difluoride (PVDF) membranes. Next, the PVDF membranes were labeled with primary antibodies (1:1000; CST Biological Reagents Company Limited, Shanghai, China) O/N at 4 °C, followed by 3× phosphate-buffered saline (PBS) washes, incubation with immunoglobulin G-horseradish peroxidase (IgG-HRP)-conjugated secondary antibodies (1:2000; Beijing Golden Bridge Biotechnology, Beijing, China) at room temperature (RT) 1 h, 3 PBS washes, and color development using chemiluminescent HRP substrate. GAPDH served as an endogenous control. All experiments were performed 3×, and the best representation of the results is presented in this paper.

### Alizarin Red S staining

The cell culture medium was discarded, and the cells were washed twice with PBS before fixation in 4% paraformaldehyde for 15 min, followed by 3 washes with double distilled water and staining in ARS solution (Beijing Solarbio Science & Technology, Beijing, China) for 30 min. Subsequently, the cells were washed again with double distilled water before observation under a light microscope. ARS staining colored the calcium nodules deep orange red.

### Immunohistochemistry

The paraffin-embedded, 4-μm-thick sections were mounted on glass slides. Next, the paraffin was removed, and the sections were hydrated to retrieve antigens before independent labeling with Sox9, Runx2 and collagen type X antibodies (CST Biological Reagents Company Limited, Shanghai, China) at a 1:200 concentration. Following washing, the sections were labeled with 1:200 goat anti-rabbit IgG-HRP secondary antibodies (Beijing Golden Bridge Biotechnology), followed by conventional color development reagent, and observed and photographed under a light microscope at 200×. Five random visual fields were selected per treatment group for analysis. Image-Pro Plus 6.0 software (Media Cybernetics, Inc., Rockville, MD, USA) was employed to analyze the stained cells in each image using the same parameters. The accumulated optical density (IOD) and the pixel area (area) of each photo were recorded, and the average IOD/area (mean density) was calculated.

### Fluorescence in situ hybridization

The sections of paraffin-embedded acetabular samples and chondrocytes were incubated with 500 ng/ml FAM-labeled probes for 48 h using a FISH kit (Shanghai Gefan Biotechnology, Shanghai, China). MiR-1-3p expression was then recorded under a fluorescence microscope and further analyzed using ImageJ (version 1.48 v).

### Statistical analysis

All data are expressed as the mean ± standard deviation. Following outlier removal, the paired *t* test was employed to compare the expression of relevant genes and proteins between DHH samples and controls. One-way analysis of variance (ANOVA) with post hoc Tukey’s test was used to compare the expression of relevant genes and proteins in chondrocytes treated with blank control, normal control, miR-1-3p mimic, si-miR-1-3p, and si-SOX9. Statistical significance was defined at a *P* value < 0.05. SPSS 25.0 software and Prism software were used to perform the statistical analyses.

## Results

### Rabbits with DDH exhibited thick ARC and low miR-1-3p expression

To examine the pathogenesis of DDH, we successfully induced DDH in 18 rabbits. Upon euthanasia, tissue specimens were collected from the hip joint of all rabbits. In particular, ARC samples from 14 left acetabular dysplasia and 14 right controls (14 rabbits with DDH) were used for RNA and protein examination, whereas ARC samples from 4 left acetabular dysplasia and 4 right controls (4 DDH rabbits) were used for HE staining, immunohistochemistry, and FISH investigation.

**Fig. 2:**
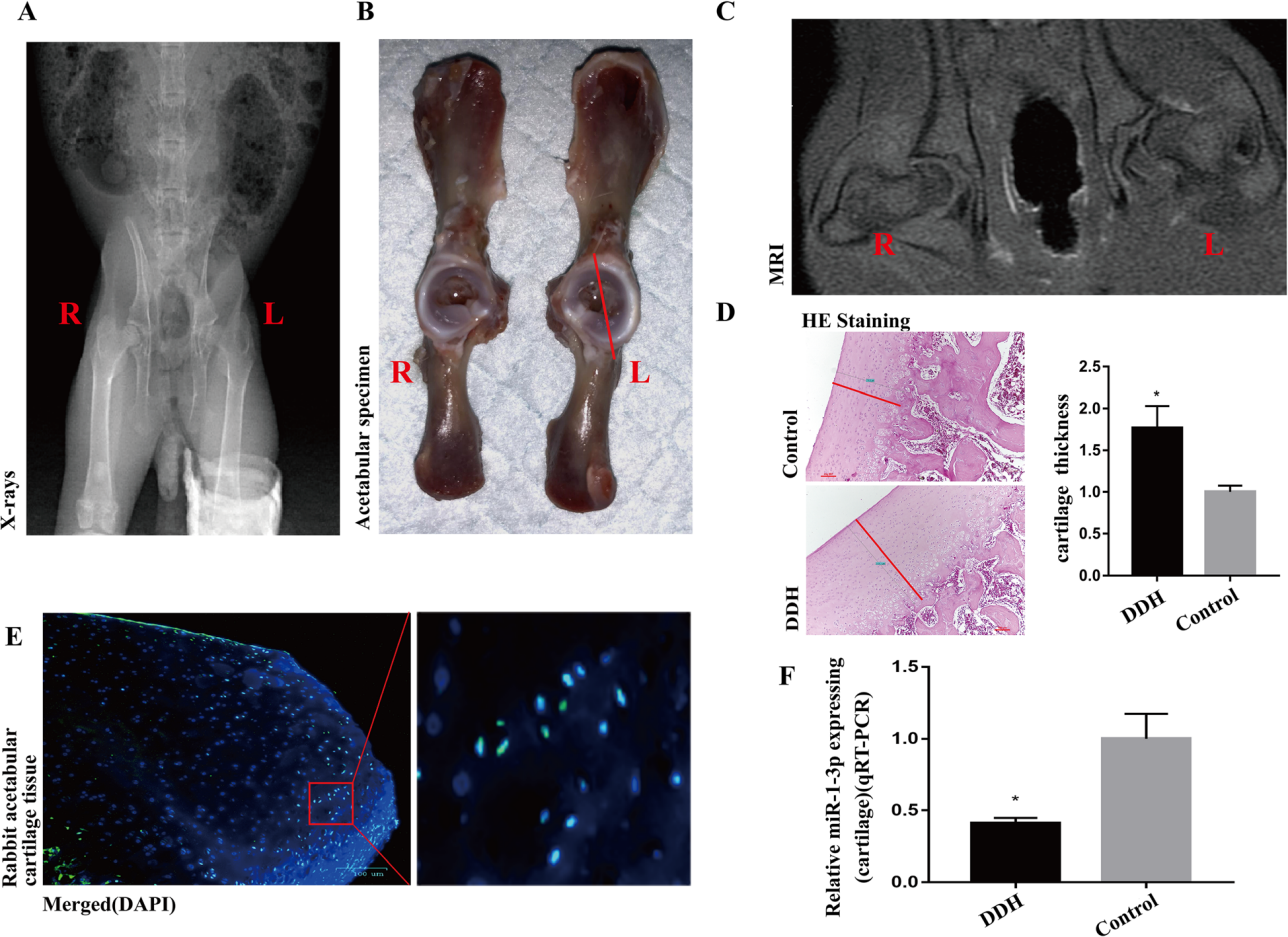
The ARC from the DDH group was remarkably thickened and had low miR-1-3p expression. **A** The successful induction of DDH in New Zealand white rabbits, as evidenced by X-ray. **B**–**D** The ARC in the DDH model was remarkably thicker than that of the untreated control limb, as evidenced by gross specimen observation, MRI imaging, and HE staining. Scale bar: 100 μm. **F** QPCR confirmed the reduced miR-1-3p expression in the DDH ARC samples versus the controls. (Data represents mean ± SD, *n* = 3. *P < 0.05.)

In this study, X-ray imaging confirmed the successful establishment of DDH in New Zealand white rabbits (Fig. 2A). Moreover, the ARC was remarkably thickened in the rabbits with DDH, as evidenced by gross specimen observation, MRI images, and HE staining (Fig. 2B–D, *P* < 0.05).

Next, we confirmed miR-1-3p expression in the acetabular roof cartilage by FISH (Fig. 2E). Subsequently, we confirmed the low expression of miR-1-3p in the acetabular roof cartilage of the DDH group compared to the healthy control group using qPCR (Fig. 2F). This result was consistent with our previously conducted miRNA chip data [13].

### Downregulation of miR-1-3p expression inhibits chondrocyte hypertrophy and reduces mineralization in vitro

**Fig. 3:**
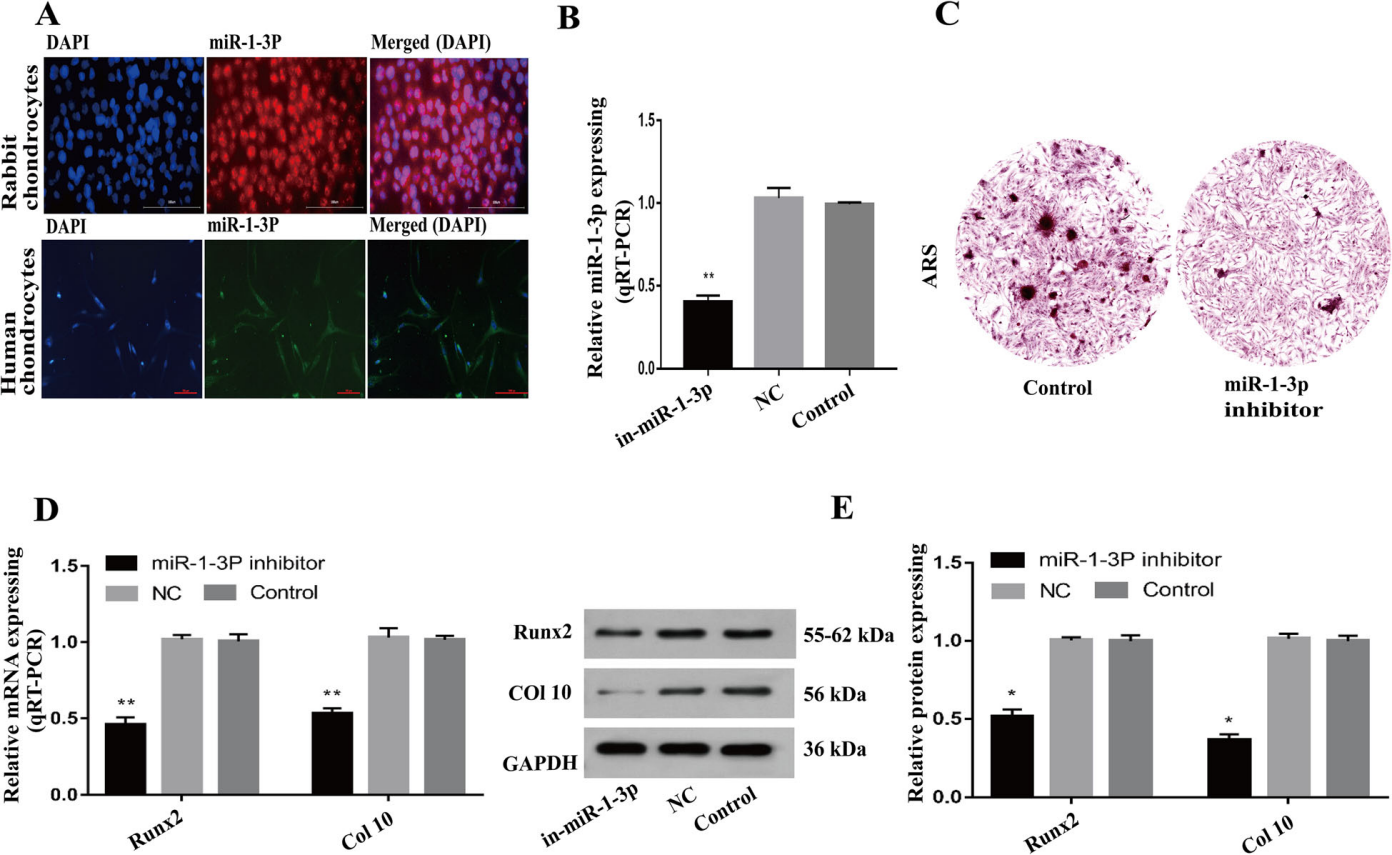
Downregulation of miR-1-3p expression suppresses chondrocyte hypertrophy and reduces mineralization. **A** FISH was used to verify miR-1-3p expression in rabbit and human chondrocytes (a). Scale bar: 100 μm. **B** Introduction of the miR-1-3p inhibitor successfully downregulated miR-1-3p levels in human chondrocytes. **C** ARS staining of the miR-1-3p-downregulated chondrocytes exhibiting reduced mineralization compared to the controls. Scale bar: 200 μm. **D**, **E** Reduced RUNX2 and collagen type X levels in the miR-1-3p-silenced chondrocytes versus the controls. (*P < 0.05, **P < 0.01)

First, we confirmed the presence of miR-1-3p in human and rabbit chondrocytes, using immunofluorescence (Fig. 3A). Next, we transfected primary human chondrocytes with a miR-1-3p inhibitor to ascertain the specific role of miR-1-3p in chondrocytes. As shown in Fig. 3B, miR-1-3p levels fell dramatically upon transfection with the miR-1-3p inhibitor (*P* < 0.01). To assess whether low miR-1-3p levels promote abnormal chondrocyte ossification, we performed ARS staining to detect calcium deposits in these cells. The miR-1-3p-inhibited cells exhibited reduced mineralization compared with the controls (Fig. 3C). We also analyzed the gene expression of the endochondral osteogenic markers RUNX2 and collagen type X and found a substantial decrease in the expression of both genes (Fig. 3D, E, *P* < 0.05).

### MiR-1-3p directly targets SOX9

**Fig. 4:**
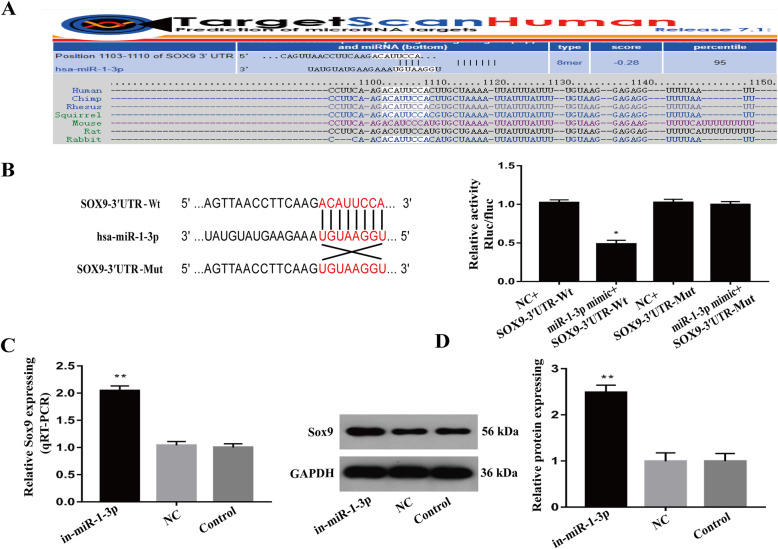
MiR-1-3p directly targets SOX9 in human chondrocytes. **A** Bioinformatics database analysis showing that miR-1-3p directly targets SOX9. **B** Luciferase reporter assay verifying the interaction between miR-1-3p and SOX9. **C**, **D** Evaluation of SOX9 transcript and protein expression in the miR-1-3p inhibitor- or NC-miRNA inhibitor-treated chondrocytes. (*P < 0.05, **P < 0.01)

Our prior bioinformatics analysis revealed SOX9 as a downstream target gene of miR-1-3p (Fig. 4A). We further confirmed this using a luciferase reporter assay in which SOX9-WT- and miR-1-3p mimic-incorporated cells showed reduced luciferase activity, but the activity was restored in the miR-1-3p mimics- and SOX9-mut-incorporated cells (Fig. 4B, *P* < 0.05). Alternately, miR-1-3p suppression using miR-1-3p inhibitors in chondrocytes resulted in dramatic increases in SOX9 transcript and protein expression (Fig. 4C, D, *P* < 0.01). These results suggest a strong modulatory relationship between miR-1-3p and Sox9.

### MiR-1-3p suppression inhibits chondrocyte hypertrophy and reduces mineralization via SOX9

**Fig. 5:**
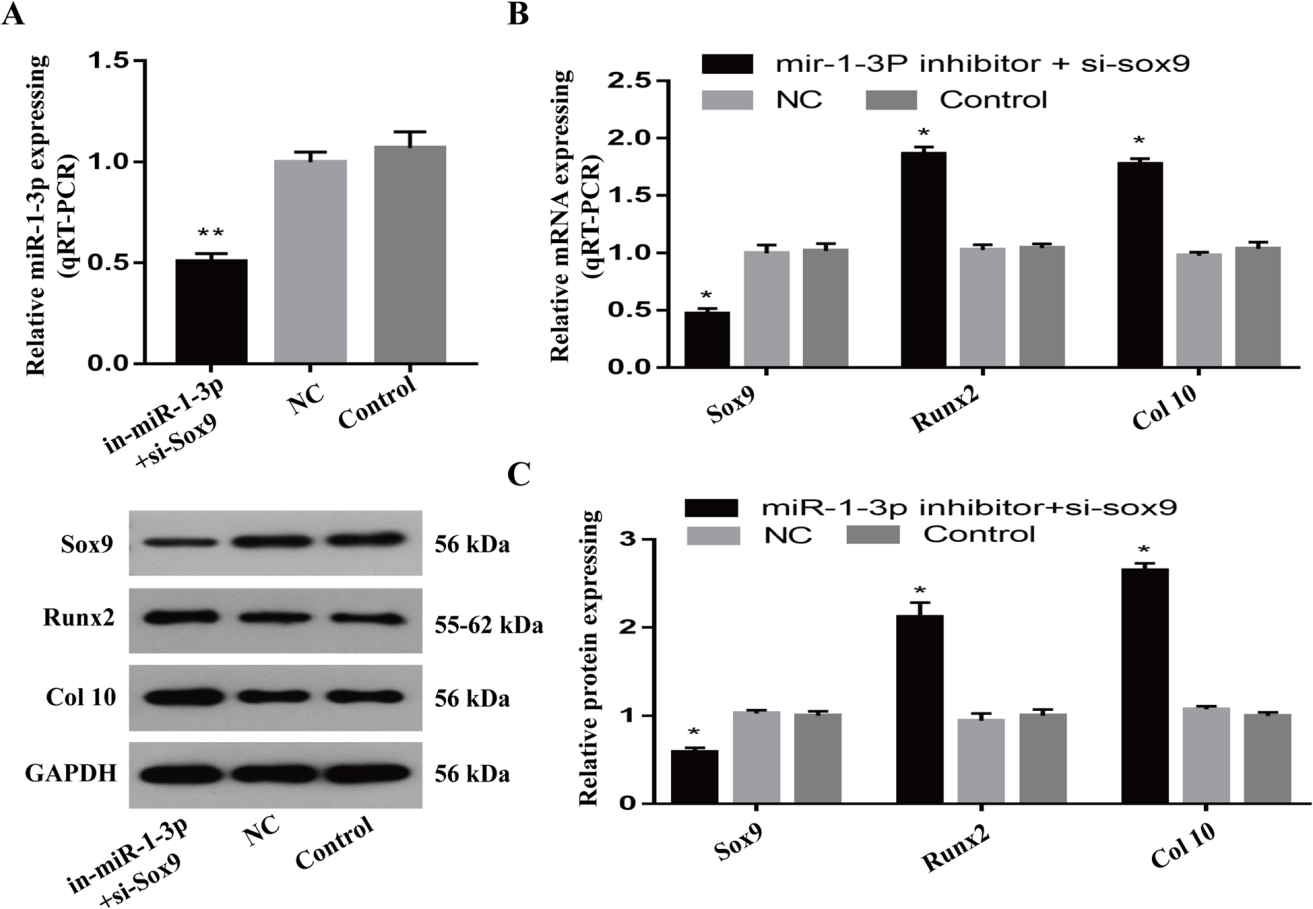
SOX9 silencing rescued the effects of low miR-1-3p expression on chondrocyte differentiation. **A** Evaluation of miR-1-3p levels after the simultaneous incorporation of miR-1-3p inhibitor and si-sox9 in chondrocytes. **B**, **C** Evaluation of SOX9, RUNX2, and collagen type X transcript and protein expression in miR-1-3p- and SOX9-silenced chondrocytes showing downregulation of SOX9 expression and simultaneous upregulation of RUNX and collagen type X expression. (*P < 0.05, **P < 0.01)

**Fig. 6:**
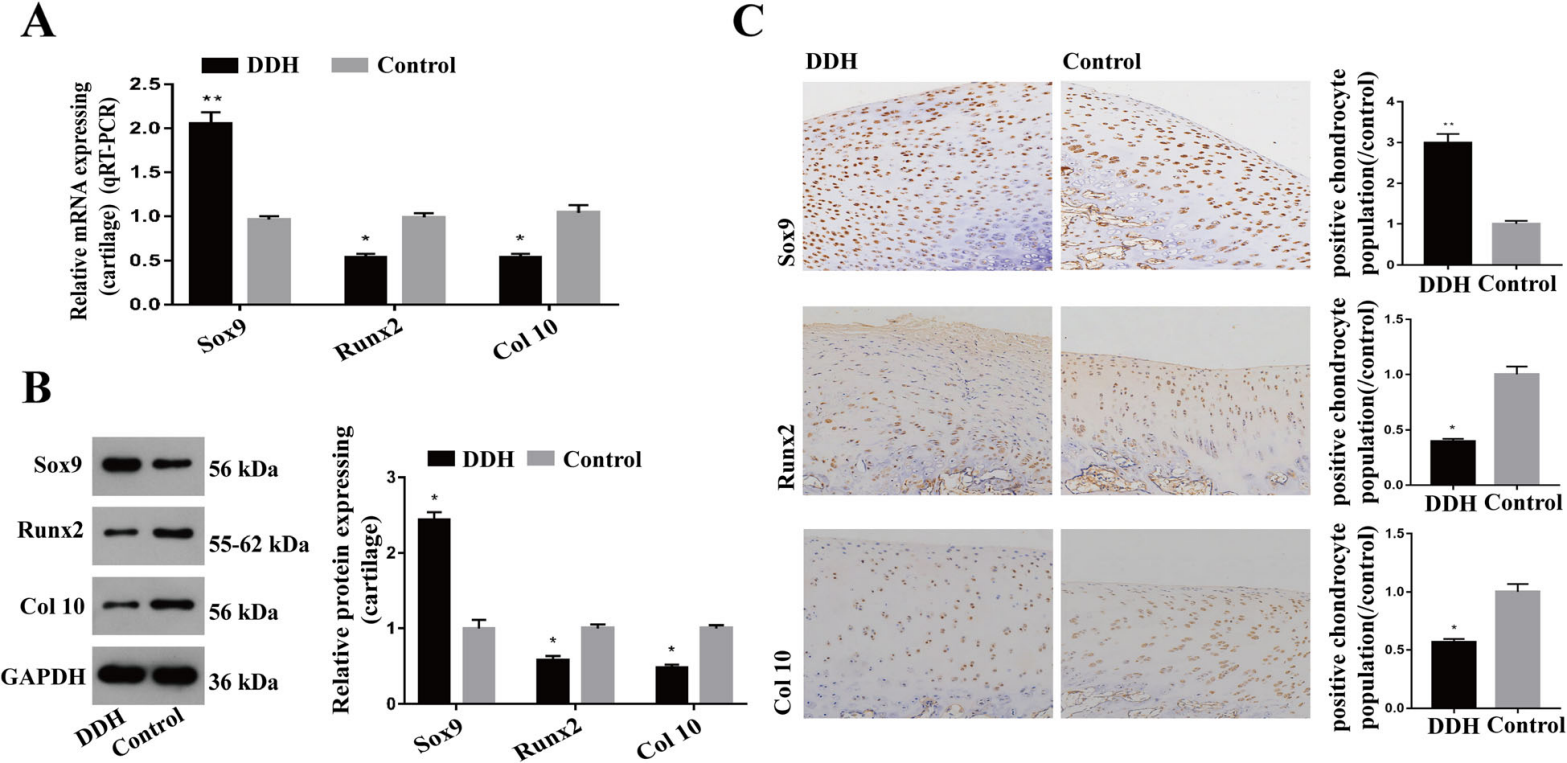
Verification of SOX9, RUNX2, and collagen type X expression in the ARC of rabbits with DHH. **A**–**C** qPCR, western blot analysis, and immunohistochemistry showed that SOX9 levels were high and RUNX2 and collagen type X levels were low in the DDH ARC specimens compared to the controls. (*P < 0.05, **P < 0.01)

Given that miR-1-3p suppression can elevate SOX9 expression and lower RUNX2 and collagen type X expression, we next examined the role of miR-1-3p in chondrocyte hypertrophy and reduced mineralization through Sox9. To do this, we simultaneously incorporated human chondrocytes with a miR-1-3p inhibitor and si-SOX9. Using RT-PCR and western blot analysis, we verified reduced expression of miR-1-3p and SOX9 in the cells, which resulted in an increased expression of RUNX2 and type X collagen transcripts and proteins compared to that of the controls (Fig. 5A–C, *P* < 0.05). Furthermore, we confirmed the high SOX9 expression and low RUNX2 and type X collagen levels in the acetabular roof cartilage of the DHH model vs healthy controls, as evidenced by immunohistochemistry, qPCR, and western blot analysis (Fig. 6A–C, *P* < 0.05).

## Discussion

Multiple reports have suggested that the increased thickness of the acetabular roof cartilage in children with DDH and animal models is associated with delayed endochondral ossification [10, 13, 23, 24]. In our previous study, we found that the MRI manifestation of the acetabular roof of rabbits with DDH is similar to the MRI manifestation from children with DDH [13]. In this study, we also observed this phenomenon, especially in terms of producing a thicker acetabular roof cartilage in DDH models (also confirmed using gross specimen inspection and HE staining).

Growing evidence suggests a crucial role of miRNAs in cartilage physiology [25]. Miyaki et al. [16], for instance, discovered the presence of miR-140 in the growth plate, which modulates the target gene Adamts-5, an important matrix protease that hydrolyses proteoglycans and type II collagen, to maintain healthy cartilage structure and function. Alternately, miR-140-knockout mice developed severe osteoarthritis. Nakamura et al. [17], however, demonstrated using miR-140-knockout mice that the Dnpep-mediated bone morphogenetic protein (BMP) signaling pathway was affected, inducing articular chondrocyte differentiation into hypertrophic chondrocytes and resulting in chondrogenic disorder. Likewise, Kobayashi et al. [18] specifically knocked out the Dicer gene in a mouse model, which dramatically reduced miRNA expression in chondrocytes and strongly suppressed bone formation and chondrocyte proliferation in the growth plate. Similarly, Zhang et al. [19] confirmed the low expression of miR-150-5p in osteoarthritis, which negatively impacted the AKT serine/threonine kinase 3 (AKT3) pathway to promote chondrocyte proliferation and inhibit apoptosis and degradation of extracellular matrix in chondrocytes. In another example, Bluhm et al. [20] reported high miR-332 expression, which modulated the RAF/MEK/ERK pathway to increase chondrocyte differentiation and promote the development of achondroplasia. Thus, miRNA modulates not only chondrocyte proliferation, differentiation, apoptosis, endochondral ossification, and cartilage development but also osteoarthritis and achondroplasia [16–20]. However, the expression and function of miR-1-3p in chondrocytes have not been elucidated, and its mechanism of action in acetabulum abnormal endochondral ossification in DDH remains unknown.

Based on existing research, miR-1-3p is a tumor-related miRNA that is involved in the viability, proliferation, and apoptosis of multiple cancerous cells. In fact, miR-1-3p expression was shown to be severely downregulated in gastric cancer, and its mechanism of action was shown to include negative regulation of STC2 to suppress cell proliferation and invasion to form gastric cancer [26]. Similarly, Gao et al. [27] reported that miR-1-3p can suppress BDNF expression and phosphorylation of TrkB to halt the proliferation and invasion of bladder cancer cells. Likewise, Zhang et al. [28] confirmed low levels of miR-1-3p in hepatocellular carcinoma cell lines, which improved Sox9 expression and cell proliferation and suppressed apoptosis in HCCLM3 and Bel-7474 cells. In addition, myogenic factors such as MyoD, Mef2, and SRF were reported to increase miR-1-3p expression, whereas skeletal muscle hypertrophy decreased miR-1-3p expression [29, 30]. Moreover, in a recent study involving Chinese patients with osteoporosis, miR-1-3p expression was reported to be significantly downregulated and SFRP1 expression was upregulated with reduced bone formation and bone mass [31]. In this study, we demonstrated low miR-1-3p levels in the ARC of the rabbits with DDH, which is consistent with the high-throughput sequencing data we obtained previously [13]. To ascertain the function of miR-1-3p in chondrocytes, we first confirmed its expression in normal human and rabbit chondrocytes using FISH. We also demonstrated that with low miR-1-3p levels, mineralized nodules also existed in these chondrocytes, as evidenced by ARS staining. Analysis of endochondral osteogenesis-related genes showed reduced expression of RUNX2 and collagen type X following miR-1-3p suppression. To identify downstream targets of miR-1-3p, we screened bioinformatics data and found SOX9. SOX9 is normally expressed in all chondrogenic progenitors and chondrocytes of the articular cartilage throughout adulthood and is a master transcription factor regulating multiple events involving chondrogenesis [32–35]. Moreover, several studies have suggested a role of SOX9 in endochondral ossification. In particular, SOX9 suppression in the normal growth plate is essential for endochondral ossification, whereas high expression of Sox9 in the growth plate retards this process [36, 37]. In accordance with other studies, we showed a significant upregulation of SOX9 expression in miR-1-3p-silenced chondrocytes in vitro. To confirm whether downregulation of miR-1-3p expression reduces RUNX2 and collagen type X expression via SOX9, we performed rescue experiments. In brief, we demonstrated that SOX9 silencing restored RUNX2 and collagen type X expression in the cells treated with miR-1-3p inhibitor versus the controls. Prior studies have reported that Sox9 negatively regulates Runx2 and type X collagen expression to modulate endochondral ossification-related disorders [37, 38]. To verify the miR-1-3p-mediated regulation of endochondral ossification genes, the levels of SOX9, RUNX2, and collagen type X were examined in the ARC of DDH and healthy rabbit acetabula using immunohistochemistry, qPCR, and western blot analysis. As expected, our results showed that low miR-1-3p levels in the ARC resulted in high expression of SOX9 and low expression levels of RUNX2 and collagen type X in the DDH rabbit acetabula versus controls. These results suggest that the low expression of miR-1-3p suppresses the expression of RUNX2 and collagen type X via the miR-1-3p/Sox9 axis, which results in delayed endochondral ossification in the acetabular roof cartilage of DDH. As a result, DDH presents increased thickness in the ARC.

## Conclusion

In summary, we demonstrated, for the first time, that miR-1-3p modulates abnormal endochondral ossification of the ARC in DDH via its regulation of the target gene SOX9. In the future, our findings need to be verified in vivo, particularly the dynamic alterations in miR-1-3p expression in the growth plate during the embryonic, neonatal, and childhood stages. We also suggest the establishment of an in vivo gene knockout model for further research in this field.

## Data Availability

Please contact author for data requests.

## Abbreviations

DDH: Developmental dysplasia of the hip
ARC: Acetabular roof cartilage
CT: Computed tomography
MRI: Magnetic resonance imaging
3DCT: Three-dimensional CT
MiRNA: MicroRNA
QPCR: Quantitative polymerase chain reaction
HE: Hematoxylin and eosin
ARS: Alizarin Red S
FISH: Fluorescence *in situ* hybridization
NC: Normal control
SOX9: SRY-Box transcription factor 9
Runx2: Runt-related transcription factor 2
Col 10: Collagen type X
